# Birt-Hogg-Dubé renal tumors are genetically distinct from other renal neoplasias and are associated with up-regulation of mitochondrial gene expression

**DOI:** 10.1186/1755-8794-3-59

**Published:** 2010-12-16

**Authors:** Jeff A Klomp, David Petillo, Natalie M Niemi, Karl J Dykema, Jindong Chen, Ximing J Yang, Annika Sääf, Peter Zickert, Markus Aly, Ulf Bergerheim, Magnus Nordenskjöld, Sophie Gad, Sophie Giraud, Yves Denoux, Laurent Yonneau, Arnaud Méjean, Viorel Vasiliu, Stéphane Richard, Jeffrey P MacKeigan, Bin T Teh, Kyle A Furge

**Affiliations:** 1 Laboratory of Computational Biology, Van Andel Research Institute, Grand Rapids, MI, USA; 2 Laboratory of Cancer Genetics, Van Andel Research Institute, Grand Rapids, MI, USA; 3 Laboratory of Systems Biology, Van Andel Research Institute, Grand Rapids, MI, USA; 4 Division of Surgical Pathology, Northwestern University Feinberg School of Medicine, Chicago, IL, USA; 5 Department of Molecular Medicine and Surgery and Center for Molecular Medicine, Karolinska Institutet at Karolinska University Hospital, SE-171 76 Stockholm, Sweden; 6 Department of Pathology, Karolinska University Hospital, SE-182 88 Stockholm, Sweden; 7 Division of Urology, Department of Clinical Sciences, Karolinska Institutet at Danderyd Hospital, SE-182 88 Stockholm, Sweden; 8 Department of Surgery, Karolinska Institutet at Danderyd Hospital, SE-182 88 Stockholm, Sweden; 9 Génétique Oncologique EPHE, INSERM U753, Le Kremlin-Bicêtre and Institut Gustave Roussy, Villejuif, France; 10 Laboratoire de Génétique, Hôpital Edouard Herriot, Hospices Civils, Lyon, France; 11 Centre de Références Cancers Rares PREDIR de l'INCa, Service d'Urologie, AP-HP, Hôpital de Bicêtre, Le Kremlin-Bicêtre, France; 12 Laboratoire d'Anatomie Pathologique, Hôpital Foch, Suresnes, France; 13 Service d'Urologie, Hôpital Foch, Suresnes, France; 14 Service d'Urologie, Hôpital Necker, Paris, France; 15 Laboratoire d'Anatomie Pathologique, Hôpital Necker, Paris, France; 16 NCCS-VARI Translational Research Laboratory, National Cancer Centre of Singapore, Singapore

## Abstract

**Background:**

Germline mutations in the *folliculin *(*FLCN*) gene are associated with the development of Birt-Hogg-Dubé syndrome (BHDS), a disease characterized by papular skin lesions, a high occurrence of spontaneous pneumothorax, and the development of renal neoplasias. The majority of renal tumors that arise in BHDS-affected individuals are histologically similar to sporadic chromophobe renal cell carcinoma (RCC) and sporadic renal oncocytoma. However, most sporadic tumors lack *FLCN *mutations and the extent to which the BHDS-derived renal tumors share genetic defects associated with the sporadic tumors has not been well studied.

**Methods:**

BHDS individuals were identified symptomatically and *FLCN *mutations were confirmed by DNA sequencing. Comparative gene expression profiling analyses were carried out on renal tumors isolated from individuals afflicted with BHDS and a panel of sporadic renal tumors of different subtypes using discriminate and clustering approaches. qRT-PCR was used to confirm selected results of the gene expression analyses. We further analyzed differentially expressed genes using gene set enrichment analysis and pathway analysis approaches. Pathway analysis results were confirmed by generation of independent pathway signatures and application to additional datasets.

**Results:**

Renal tumors isolated from individuals with BHDS showed distinct gene expression and cytogenetic characteristics from sporadic renal oncocytoma and chromophobe RCC. The most prominent molecular feature of BHDS-derived kidney tumors was high expression of mitochondria-and oxidative phosphorylation (OXPHOS)-associated genes. This mitochondria expression phenotype was associated with deregulation of the PGC-1α-TFAM signaling axis. Loss of *FLCN *expression across various tumor types is also associated with increased nuclear mitochondrial gene expression.

**Conclusions:**

Our results support a genetic distinction between BHDS-associated tumors and other renal neoplasias. In addition, deregulation of the PGC-1α-TFAM signaling axis is most pronounced in renal tumors that harbor *FLCN *mutations and in tumors from other organs that have relatively low expression of *FLCN*. These results are consistent with the recently discovered interaction between FLCN and AMPK and support a model in which FLCN is a regulator of mitochondrial function.

## Background

Renal cell carcinomas (RCC) represent the most common type of tumors that arise within the adult kidney. They can be divided into several subtypes - clear cell, papillary, chromophobe, and collecting duct - based on differences in cellular morphology, gene expression, and cytogenetic and genetic abnormalities that are found within the tumor cells [1-4]. The two most common types of RCC are clear cell and papillary, which together account for approximately 85-90% of RCCs. Chromophobe RCC accounts for an additional 5% of renal tumors, and a histologically similar subtype, renal oncocytoma, represents another 5% (see [5,6] for recent reviews). Although the neoplastic cells of chromophobe RCC and renal oncocytoma share morphological features, renal oncocytomas are generally asymptomatic and nearly always present as localized lesions with low metastatic potential [7].

Though most renal tumors occur sporadically (~95%), several hereditary syndromes are associated with a high risk of renal tumor development. These syndromes include von Hippel-Lindau disease, hereditary papillary RCC, hereditary leiomyomatosis and renal cancer, and Birt-Hogg-Dubé syndrome (BHDS) [8]. In von Hippel-Lindau disease, a rare germline mutation in the *VHL *gene is associated with development of the disease (reviewed in [9]). Individuals with von Hippel-Lindau disease are predisposed to the development of renal tumors of the clear cell histology. In addition, somatic mutations in the *VHL *gene are also found in the majority of the sporadic cases of clear cell RCC [10]. Birt-Hogg-Dubé syndrome is an extremely rare syndrome-approximately 200 families have been described as having BHDS worldwide [11,12]. Germline inheritance of a mutated allele of the *folliculin *(*FLCN*) gene, located at chromosome location 17p11.2, is strongly associated with individuals that develop BHDS [13]. In individuals afflicted with BHDS, the majority (~85%) of renal tumors that develop are histologically similar to chromophobe RCC or described as oncocytic hybrid tumors, with portions appearing as both renal oncocytoma and chromophobe RCC [14,15]. Unlike *VHL*, somatic mutations in the *FLCN *gene are not strongly associated with the development of sporadic renal oncocytoma and chromophobe RCC [16,17]. As such, the role that *FLCN *plays in the development of sporadic renal oncocytoma, chromophobe RCC, and other sporadic tumors remains unclear.

The folliculin gene encodes a highly conserved, 64kD protein with no known functional domains. Recent reports support its role as a tumor suppressor [18,19] and in energy-related signaling, involving the mTOR and AMPK pathways [20-22]. FLCN has been shown to interact with AMPK through the binding of two intermediary proteins, folliculin interacting protein 1 and folliculin interacting protein 2 (FNIP1/2) and the activity of FLCN may be altered by its subsequent phosphorylation by AMPK or localization to the cytoplasm with its binding partners, or a combination of these two mechanism [20,23,24]. As indicated previously, while germline mutations in *FLCN *cause BHDS, these mutations are not strongly associated with either sporadic chromophobe RCC or renal oncocytoma [17]. The most well characterized somatic mutations found in these two sporadic tumor subtypes are mutations within the mitochondrial genome [25-29]. Renal oncocytoma, in particular, is characterized by the accumulation of somatic mutations in mtDNA that inactivate subunits of mitochondrial complex I and other members of the electron transport chain, severely limiting ATP production[26,27]. In addition, both sporadic renal oncocytoma and chromophobe RCC possess mitochondria-dense cytoplasm and aberrant expression of genes associated with oxidative phosphorylation (OXPHOS) [25,27,30]. However, the mechanism by which these mitochondrial defects contribute to tumor development remains unclear and the gene expression and cellular phenotypes observed are thought to represent feedback mechanisms to compensate for mitochondrial impairment.

While expression of some key markers of renal tumors have been examined in a single BHDS-derived tumor [31], we conducted gene expression profiling of multiple renal tumors that arose in individuals with BHDS along with sporadic renal oncocytoma and chromophobe RCC to develop a better understanding of the underlying molecular genetics of these tumors. We found that tumors that arose in individuals with BHDS were genetically distinct from sporadic tumors, showing distinct gene expression and cytogenetic characteristics. However, similar to sporadic renal oncocytoma and chromophobe RCC, BHDS-derived renal tumors displayed high expression of mitochondria and OXPHOS-associated genes. Indeed, the expression of mitochondria and OXPHOS-associated genes was even more pronounced in the BHDS-derived tumors than the other sporadic tumors and was correlated to increased expression of key mitochondria transcriptional regulators. We have also noted an inverse correlation between *FLCN *expression and mitochondria- and OXPHOS-associated genes across a variety of tumor types, most evident in tumors that possessed relatively low levels of FLCN and enrichment in mitochondria- and OXPHOS-associated gene expression. Taken together, our data suggest that FLCN has an important role in the regulation of genes associated with mitochondria and OXPHOS in BHDS-derived tumors and possibly others.

## Methods

### Tissue sample collection and DNA sequencing

Internal review board approval was obtained from each participating institution for the renal neoplasms under study. Samples isolated from individuals afflicted with BHDS were flash-frozen in liquid nitrogen and stored at -80°C following excision from patients as previously described [32]. *FLCN *mutation status was confirmed through DNA extraction from tumor samples and sequencing, as described previously [33], using primer sequences from Nickerson *et al*. [13]. The histological classification and *FLCN *mutation information for the BHDS-derived renal tumor samples are given in Additional file 1 Table S1.

### Gene expression profiling datasets

RNA was isolated and expression profiles generated from BHDS-derived tumor samples using the Affymetrix HG-U133 Plus 2.0™chipset as previously described [32]. These data are available at the Gene Expression Omnibus (GEO, GSE21816). Expression profiles for the remaining RCC subtypes and non-RCC tumors used in the analysis are publicly available from the GEO database (GSE8271, GSE11024, GSE11016, GSE7023, and GSE2109). All data analysis was performed using software available from the BioConductor Project (*version 2.5*) and the R statistical environment v. 2.10.1 [34,35]. Prior to analysis, the robust multi-chip average (RMA), as implemented in the A*ffy *package (1.24.2), was used for background correction and normalization of raw expression image intensities using updated probeset mapping [36] and data were normalized to corresponding normal tissue type. The technical replicate expression datasets from the DT017 sample of patient BHD1 were averaged prior to discriminate gene and gene set analyses.

### Validation of gene expression microarray data by qRT-PCR

Single-step, quantitative reverse transcription-PCR (qRT-PCR) was performed to validate expression levels for the following genes: *PVALB, CDH19, RGS20*, and *LRRTM4*, with the *GAPDH *gene as a control. To perform the single-step qRT-PCR, we used the Power SYBR^® ^Green PCR Master Mix with Taqman^® ^Gold RT-PCR enzymes (Applied Biosystems, Foster City, CA). We also conducted qRT-PCR using Taqman^® ^assays (Applied Biosystems) using the manufacturer's protocol for the following genes: *FLCN, FNIP2, PPARGC1A, PVALB, RGS20, TFAM, and TSC1*. The reactions were run on an ABI 7500 Fast Real-Time PCR system using a dissociation curve analysis for the SYBR Green assays to confirm primer specificity. We used the *PerlPrimer *software [37] to design PCR primers within the exons that were interrogated by the Affymetrix expression chips. Primer sequences and assay ids have been made available in Additional file 1 Table S4.

### Clustering and differential gene expression

Prior to clustering of all RCC samples, the 1000 most variable genes were isolated using an interquartile range filter of greater than 1.54. Clustering was performed using Euclidean distance with complete linkage. For the clustering of sporadic chromophobe RCC, sporadic oncocytoma, and BHDS-derived renal tumor samples, the 1500 most variable genes were isolated, corresponding to an interquartile range filter of greater than 0.79. Euclidean distance with average linkage was used, followed by resampling for node support. Bootstrap resampling for 10,000 replications and a relative sample size of 1 was used to generate the bootstrap probability values, as implemented in the *pvclust *package v.1.2-1 [38].

Discriminatory genes were identified using a moderated t-statistic as implemented in the *limma *package. Significance values were adjusted to correct for multiple testing using the Benjamini and Hochberg method [39]. Genes with false discovery rate (FDR) values less than 0.01 were reported as significant. Given that the sample size of BHDS-derived tumors was disproportionate to the number of either sporadic oncocytoma or chromophobe RCC tumors, we conducted a permutation test to decide whether the distinctiveness of BHDS-derived tumors was a result of bias from a sample size effect. The test was conducted using 1000 iterations comparing the entire data set from the six BHDS-derived tumors to five randomly selected oncocytoma data sets (without replacement). The number of significantly differentially expressed genes from this BHD-oncocytoma comparison was greater than the number derived from a similar discriminate analysis of five randomly selected oncocytoma data sets with the remaining six oncocytoma data sets in all of 1000 permutations. Likewise, a similar permutation test using the six BHD and six randomly selected chromophobe RCC datasets was found to contain a greater number of differentially expressed genes than a comparison of six randomly chosen chromophobe with the remaining six chromophobe datasets in all of 1000 permutations.

### Gene set enrichment analyses

Parametric gene set enrichment was used to identify chromosomal expression abnormalities using gene sets corresponding to chromosomal arms as implemented in the *reb *package [40]. For pathway analysis 1892 gene sets were obtained from the Molecular Signatures Database v2.5 (MsigDB, http://www.broadinstitute.org/gsea/msigdb/). These gene sets were curated from multiple sources including online pathway databases, biomedical literature, and mammalian microarray studies. Parametric gene set enrichment analysis method as implemented in the *PGSEA *package was used generate enrichment scores for each pathway within each tumor sample using corresponding non-diseased kidney tissue as a reference. A moderated t-statistic as implemented in the *limma *package [41] was used to identify gene set enrichment scores that could discriminate between subtypes. In order to visualize the fraction of genes that overlapped between deregulated gene sets, we calculated pair-wise dissimilarity (D) scores using the formula: $D=-1+\frac{N_{A\cap B}}{2}\left(\frac{1}{N_{A}}+\frac{1}{N_{B}}\right)$, where *N*_*A*∩*B *_is the number of genes in common between gene sets A and B and *N*_*A *_and *N*_*B *_are the numbers of genes making up gene sets A and B. The dissimilarity score was used to compute a hierarchical clustering dendrogram using Euclidean distance with average linkage.

### PGC-1α signature generation

We produced a gene overexpression signature of PGC-1α using gene expression data obtained from the comparison of PGC-1α transfected HepG2 cells to mock transfected cells (GSE5968). A moderated t-statistic was used to identify genes with expression differences that were both significant (FDR < 0.00001) and had a greater than two-fold change in expression (N = 374).

## Results

### BHDS tumors have distinct gene expression patterns

**Figure 1:**
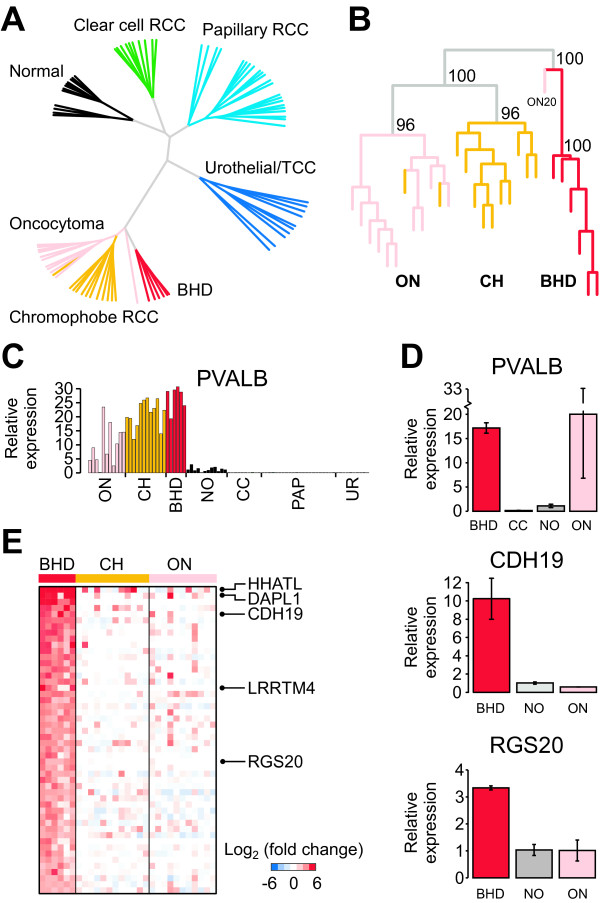
**BHD tumors represent a distinct class of renal cell carcinoma**. A) Hierarchical clustering of renal tumor samples (BHDS, N = 6; ON, N = 11; CH, N = 12; CC, N = 10; PAP, N = 22; UR, N = 10) and non-diseased renal tissue (N = 12) using the expression data from the 1000 most variable genes. B) Unsupervised clustering of BHD, ON, and CH tumor samples using gene expression data from the 1500 most variable genes within this group. Bootstrap probability values are given for the major nodes. C) Expression of the distal convoluted tubule marker *parvalbumin*, *PVALB*, in the RCC tumor sample data used in A. D) qRT-PCR validation of expression of *PVALB*, along with two identified genes with high BHDS tumor-specific expression, *cadherin 19 *(*CDH19*) and *regulator of G-protein signaling 20 *(*RGS20*). BHD, N = 2; CC, N = 3; NO, N = 3; ON, N = 3. E) Gene expression heatmap displaying expression values after median centering for the fifty genes most up-regulated in BHDS-derived tumors compared to sporadic chromophobe RCC and renal oncocytoma from A. Abbreviations: NO, normal; ON, renal oncocytoma; CH, chromophobe RCC; CC, clear cell RCC; PAP, papillary RCC; UR, urothelial/TCC RCC.

Although BHDS is exceedingly rare, it is important to determine whether molecular analysis of BHDS-derived renal tumors could give insight into the development of sporadic chromophobe RCC and renal oncocytoma as well as the cellular role of FLCN-related signal transduction. Therefore, we performed gene expression profiling on a set of renal tumors isolated from individuals afflicted with BHDS. We confirmed the presence of *FLCN *mutations in these tumors (Additional file 1 Table S1). To determine how the BHDS-derived renal tumors were related to other subtypes of renal cell carcinomas, we used unsupervised hierarchical clustering with the most variable set of expressed genes (Figure 1A). Sporadic renal oncocytoma and chromophobe RCC have an overall distinct pattern of gene expression relative to other RCC subtypes and consistent with the previously described histological similarity, the expression characteristics of BHDS-derived tumors were more similar to sporadic chromophobe and renal oncocytoma than the other RCC subtypes (Figure 1A). Sporadic renal oncocytoma and chromophobe RCC are thought to arise from cells that make up the distal convoluted tubule (DCT) portion of nephrons within the kidney [1]. To examine the tissue of origin of the BHDS-derived tumors, we assessed the expression of the distal convoluted tubule marker, *PVALB *[42]. This gene is expressed in sporadic renal oncocytoma and chromophobe RCC, but is absent or significantly lower in gene expression array data of clear cell and papillary tumors thought to derive from the proximal convoluted tubule and the urothelial/transitional cell carcinomas that arise from cells of the urinary tract (Figure 1C). Although not noted earlier, *PVALB *is highly expressed in the BHDS-derived tumors, supporting the notion that these tumors also arise from the distal convoluted tubule[31]. We further examined *FLCN *expression in BHDS-derived tumors as well as renal oncocytoma and chromophobe RCC. We did not find a significant difference in the *FLCN *transcript levels in these tumors by the gene expression array data nor by qRT-PCR of a subset of samples (Additional file 2, Figure S1A and data not shown).

In the initial gene expression analysis the BHDS-derived tumors formed a distinct branch in the clustering diagram (Figures 1A, B). These gene expression differences were not due to a sample batch effect since these renal tumors were collected at multiple institutions and the gene expression profiles were generated at various times between 2004 through 2009 using multiple chip lots (Additional file 1, Table S1 and data not shown). A more focused examination of the DCT-derived tumors confirmed those from patients with BHDS possess distinct expression characteristics with strong node support as inferred by gene resampling (Figure 1B). Several genes were differentially expressed between BHDS-derived tumors and renal oncocytoma (n = 401) and BHDS-derived tumors and chromophobe RCC (n = 2922; FDR < 0.01; Additional file 1, Table S2). For comparison, we found 1050 differentially expressed genes between sporadic oncocytoma and chromophobe RCC. Moreover, we saw few, if any, gene differences when we performed resampling with the discriminate analysis within either the sporadic renal oncocytoma or sporadic chromophobe samples, indicating the high numbers of differentially expressed genes between tumor subtypes were not due to differences in sample size between the tumor subtypes (p < 0.001, see Methods). The molecular distinction between BHDS-derived tumors, sporadic renal oncocytoma, and sporadic chromophobe RCC is in contrast to the similarities of VHL disease-associated tumors with sporadic clear cell RCC. In those studies, no significant differences in gene expression were identified between the two entities [43]. Together, the gene expression analyses indicate that distinctions exist between BHDS-derived renal tumors and other RCC subtypes similar in magnitude to those between the other recognized subtypes of RCC, such as oncocytoma and chromophobe RCC. Notable genes that are more highly expressed in BHDS-derived tumors when compared to sporadic renal oncocytoma and chromophobe RCC include *CDH19*, *RSG20*, *DAPL1*, *LRRTM4*, and *HHATL *(Figure 1EAdditional file 2, Figure S2, and Additional file 1, Table S2). We validated the expression levels of *PVALB *and three of the most significantly over-expressed genes, *CDH19 *(*cadherin 19, type 2*), *RGS20 *(*regulator of G-protein signaling 20*), and *LRRTM4 *(*leucine rich repeat transmembrane neuronal 4*) using qRT-PCR (Figure 1DAdditional file 2, Figures S1B-C, and data not shown). We chose to validate these particular genes for their consistently high expression in BHD-derived tumor samples, their low expression in the other RCC subtypes examined.

### BHDS-derived tumors lack evidence of cytogenetic features present in sporadic oncocytoma and chromophobe RCC tumors

**Figure 2:**
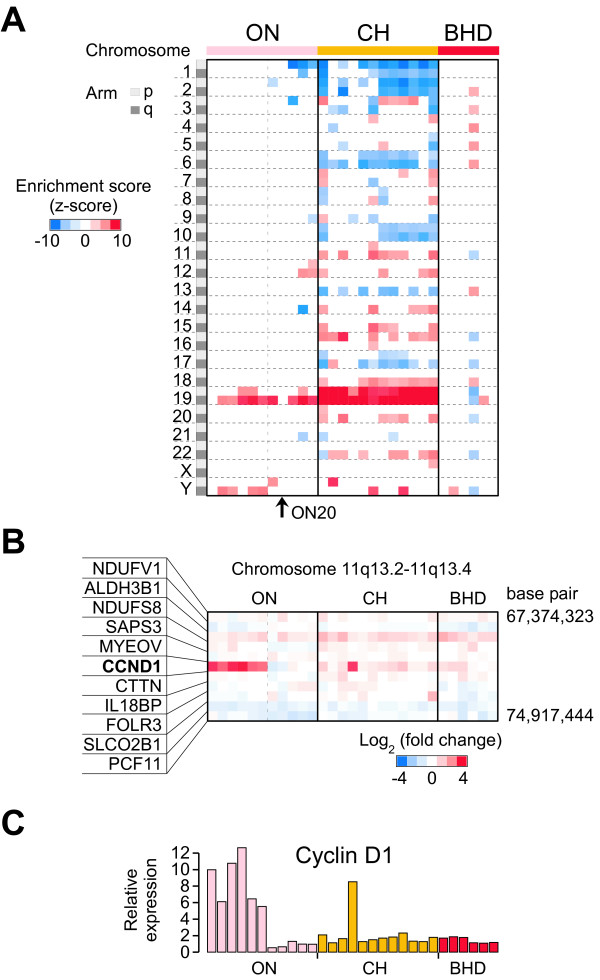
**BHD tumors do not share the cytogenetic features of sporadic chromophobe RCC and renal oncocytoma**. A) CGMA plot of BHD, CH, and ON tumor samples (columns) and chromosomal arms (rows) (p < 0.001). Blue indicates regions with a predicted copy number loss while red indicates regions with a predicted copy number gain or somatic chromosome pairing. The vertical dashed line separates oncocytoma samples with high *cyclin D1 *(*CCND1*) expression on the left from those with low expression on the right. B) Heatmap showing expression values for *CCND1 *and neighboring genes on chromosome 11q, with sample columns aligned as in part A. C) Relative gene expression for *CCND1*, with samples arranged as given in the columns of the previous parts A and B.

Several studies have shown that is possible to detect both chromosomal translocations[44] and gains and losses of large chromosomal regions through examination of gene expression data [45]. To identify potential chromosomal abnormalities that exist in BHDS samples, we examined the gene expression data for chromosome-based changes in gene expression that reflect cytogenetic changes such as chromosomal amplifications or deletions [41,45]. As with previous cytogenetic studies, our analysis predicted losses of chromosomes 1, 2, 6, 10, and 17 in chromophobe RCC and, with the exception of chromosome 1, a lack of large chromosomal abnormalities in renal oncocytoma samples (Figure 2A) [46]. In addition, evidence of a recently described abnormality of chromosome 19 (chromosomal gains and somatically paired chromosomes) was also apparent in both chromophobe RCC and renal oncocytoma data [47]. Though we predicted one BHDS-derived tumor sample (BHD4, Additional file 1, Table S1) contains multiple abnormalities involving chromosomes 2, 3, 4, 5, 6, 13, and 18, a phenomenon that is sometimes observed in sporadic cases of renal oncocytoma [48], the tumor possessed histology typical of hybrid oncocytic-chromophobe BHDS-derived tumors (Additional file 2, Figures S3A-B). The BHDS-derived tumors appeared mostly devoid of chromosomal abnormalities that are typical of the sporadic tumors. Although the BHDS-derived tumors did not show loss of chromosome 17p as described in a cell line recently established from a renal cell carcinoma of a patient with BHDS [49], the resolution of this approach does not allow us to exclude the presence of small focal deletions. In addition, sporadic renal oncocytomas can be partitioned into two mutually exclusive groups based on cytogenetic features. One group of tumors possesses a loss of chromosome 1 and the other group of tumors has a translocation of chromosome 11q13 that has a breakpoint proximal to the *cyclin D1 *(*CCND1*) gene [50]. Consistent with this finding, we identified a subgroup of renal oncocytomas with high *CCND1 *expression (N = 6, Figures 2B, C) that were independent of renal oncocytomas with a predicted loss of chromosome 1 (Figure 2A). None of the BHDS-derived tumors show evidence of the *CCND1 *associated translocation of 11q13 or loss of chromosome 1. Taken together, differences in the overall gene expression profiles and differences in predicted chromosomal abnormalities suggest that BHDS-derived renal tumors represent a genetically distinct type of renal tumor.

### A mitochondrial gene expression phenotype is a prominent feature of BHDS-derived tumors

**Figure 3:**
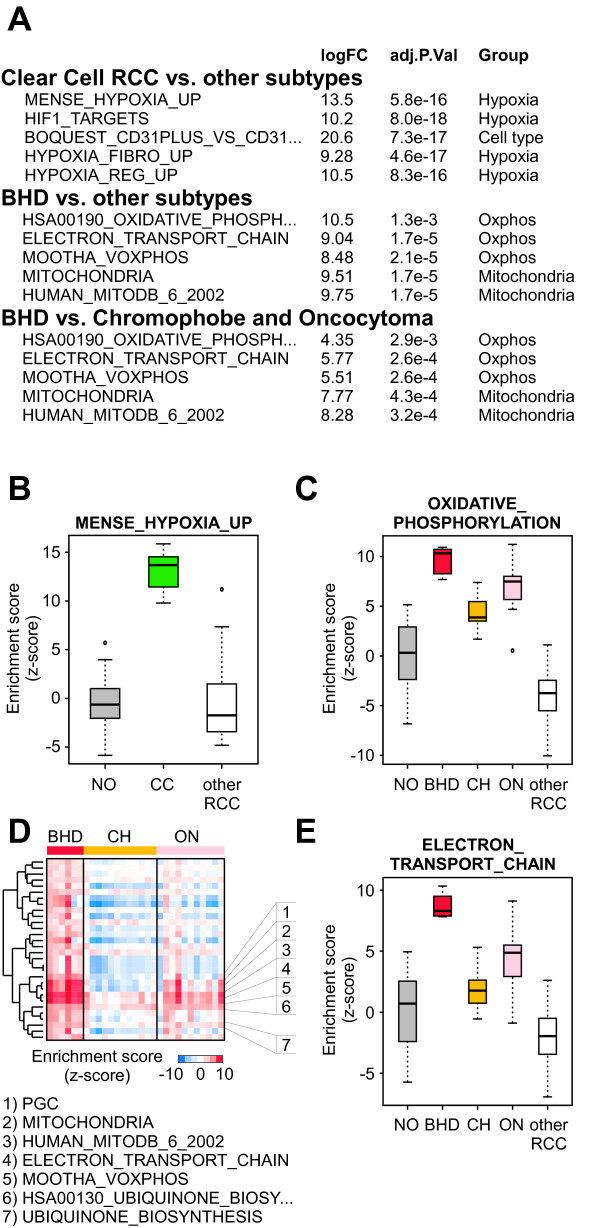
**A mitochondrial phenotype is the most prominent molecular feature of BHDS renal tumors**. A) Top differentially expressed gene sets in CC and BHD tumors. The first and second groups represent those gene sets from MsigDB that are unique to CC and BHD, respectively, as compared to the other RCC subtypes given in Figure 1A. The third group is a comparison of the BHD tumor gene expression data with only CH and ON gene expression data. B) The most differentially expressed gene set in CC tumor samples compared to the other RCC subtypes. C) The most differentially expressed gene set in BHDS tumor samples compared to the other RCC subtypes. D) Heatmap of enrichment scores for the thirty most differentially expressed gene sets in BHD versus CH and ON. On the left is a dendrogram displaying the calculated pairwise distances between dissimilarities of gene set compositions. E) The most differentially expressed gene set in BHD tumors as compared with CH and ON. Note that enrichment values for the other RCC subtypes are given here for reference in B, C, and E.

The deregulation of signal transduction pathways have been identified through examining gene expression data of renal tumors in several cases, including the deregulation of VHL, MYC, PI3K, E2F, and OXPHOS in clear cell, papillary, transitional cell carcinoma of the renal pelvis, Wilms' tumor, and renal oncocytoma, respectively [51-54]. For example, inactivation of the *VHL *gene by somatic mutation is a common feature of clear cell subtype of RCC. Cells that lack a functional VHL protein are unable to degrade the hypoxia inducible transcription factor (*HIF*). As a consequence these cells have uncontrolled expression of genes controlled by the HIF transcription factor. When parametric gene set enrichment analysis (PGSEA) is used in conjunction with gene sets (n = 1892) obtained from the Molecular Signatures Database (MSigDB, see Methods), four of the top five most significantly deregulated pathways unique to the clear cell RCC subtype were associated with a cellular hypoxia phenotype (Figure 3A, B). In a similar comparison of BHDS-derived tumors with the other RCC subtypes, the top five most significantly deregulated pathways were associated with OXPHOS or mitochondria (Figure 3A, C). This result is consistent with the high mitochondria and OXPHOS-associated gene expression observed in both sporadic oncocytoma and chromophobe RCC, tumors known to contain an abundance of mitochondria. In this regard, BHDS-derived tumors are similar to the other sporadic DCT-derived tumors. Since our analyses of individual gene expression supported distinctions between BHDS-derived tumors and sporadic renal oncocytoma and chromophobe RCC, we used PGSEA to assess whether any gene sets were uniquely enriched in BHDS-tumors. For clarity in presentation, we have organized these differentially expressed gene sets by hierarchical clustering based on the percentage of overlapping genes within gene sets (see Materials and Methods). In this way, gene sets that were highly redundant (*i.e*. contained a large percentage of overlapping genes) were located within the same branch of the clustering dendrogram. Somewhat surprisingly, several gene sets that were associated with mitochondrial function were also identified as being significantly up-regulated in BHDS-derived tumors when compared to sporadic renal oncocytoma and chromophobe RCC (Figure 3D, E). These enriched gene sets of the BHDS-derived tumors included two hand-curated gene sets reflective of peroxisome proliferator-activated receptor γ coactivator 1α (PGC-1α, encoded by the *PPARGC1A *gene) activation, MOOTHA_VOXPHOS and PGC[55]. A full list of the pathways most deregulated in BHDS-derived tumors is included as Additional file 1, Table S3.

### An expression phenotype involving the PGC-1α-TFAM signaling axis is unique to BHDS-derived tumors

**Figure 4:**
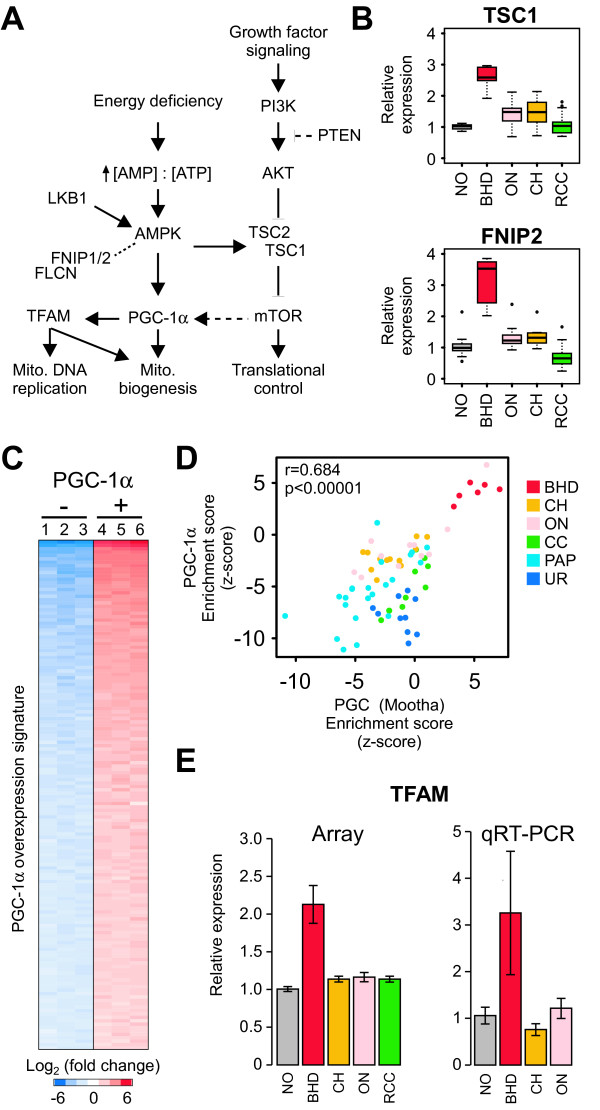
**BHDS-derived tumors possess characteristics of an active PGC-1α-TFAM signaling axis**. A) Schematic of FLCN interacting proteins in signal transduction pathway. B) Relative gene expression levels for *tuberous sclerosis 1 *(*TSC1*) and *folliculin interacting protein 2 *(*FNIP2*) proteins in tumors from patients with BHDS, ON, CH, and the other RCC subtypes from Figure 1A. C) An independent PGC-1α signature from over-expression of *PGC-1α *in HepG2 cells (*GSE5968*), showing the top 150 genes (rows) that are up-regulated in *PGC-1α *over-expressing cells compared to controls (columns). Red indicates high expression and blue indicates low expression. D) Correlation of empirically-derived *PGC-1α *signature represented in C compared to the PGC signature from Figures 3A and 3D, applied to the six RCC subtypes, using Pearson's correlation. E) Relative expression of the *TFAM *transcription factor involved in mitochondrial biogenesis (all p ≤ 0.01) in gene expression array data from BHD, CH, ON, and the remaining RCC subtypes, as well as non-diseased tissue and from qRT-PCR validation of a subset of those samples.

The presence of *FLCN *mutations in BHDS-derived tumors suggested we might be able to identify signal transduction events associated with FLCN function (Figure 4A). Previous studies of the *FLCN *gene product have indicated a role for this protein in regulation of 5' AMP-activated protein kinase (AMPK) and activation of the mTOR signalling pathway. Specifically, FLCN forms a complex with folliculin interacting protein 1 or 2 (FNIP1 or FNIP2) and the FLCN-FNIP complex binds to AMPK [20,23,24]. When we examined twelve genes encoding the proteins described in Figure 4A (*AKT1, FLCN, FNIP1, FNIP2, PIK3C3, PPARGC1A, PRKAA2, RICTOR, RPTOR, TFAM, TSC1*, and *TSC2*) in our gene expression array data, we noticed a slightly elevated level of *FNIP1 *expression in BHDS-derived tumors (data not shown) and that *FNIP2 *was highly deregulated in BHDS-derived tumors, suggesting that these proteins are relevant to FLCN signaling in renal tumor cells (Figure 4BAdditional file 2, Figure S1D). While FNIP1 and FNIP2 share a C-terminal protein domain that binds FLCN, their respective N-terminal domains are quite dissimilar and it is speculated that these proteins have non-redundant functions [23,24]. In addition, consistent with deregulation of the mTOR pathway, we also noted the deregulation of *TSC1*, a major regulator of mTOR, in the BHDS-derived tumors (Additional file 2, Figure S1E).

We also examined transcription levels of genes associated with AMPK signaling, as this was a likely candidate for signaling based on our observation of mitochondrial gene set enrichment and the recently discovered indirect interaction between FLCN and AMPK. AMPK is a key molecule for energy sensing and a regulator of the PGC-1α transcription factor, a potent inducer of mitochondrial biogenesis (Figure 4A). We noted that two transcription factors, *PGC-1α *and *TFAM *(*transcription factor A, mitochondrial*), were also up-regulated in the BHDS-derived tumors (Figure 4E and Additional file 2, Figure S1G). Both transcription of mitochondrial genes and replication of the mitochondrial genome depend on TFAM function and the *TFAM *gene is uniquely over-expressed in the BHDS-derived tumors (for a review of transcriptional regulators of mitochondria, see [56-58]). *PGC-1α (PPARGC1A) *was also highly expressed in the BHDS-derived tumors as measured by gene expression profiling. However, the levels of *PGC-1α *as measured by qRT-PCR in BHDS tumors were sensitive to the probe/primer sets used, suggesting that BHDS tumors may have a difference in the abundance of a particular *PGC-1α *isoform (Additional file 2, Figure S1G). The *PGC-1α *binding partner, nuclear receptor *peroxisome proliferator-activated receptor gamma *(*PPARG*) was highly expressed in BHDS-derived tumors as compared to non-diseased tissue, sporadic oncocytoma, and chromophobe RCC (Additional file 2, Figure S1F) while the *peroxisome proliferator-activated receptor alpha *(*PPARA*) was higher in BHDS-derived tumors versus sporadic oncocytoma and chromophobe (data not shown). Moreover, we found a set of *PGC-1α *regulated genes, entitled "PGC," was highly up-regulated in BHDS-derived samples (Figure 3D). To confirm this "PGC" gene set from MsigDB was representative of PGC-1α activation, we generated an independent gene expression signature from HepG2 cells that were adenovirally infected with PGC-1α versus control (Figure 4C, performed by Gaillard *et al*.) [59]. Although there was only 11.8 percent similarity between these two independently generated PGC-1α gene sets, both gene sets were significantly up-regulated in BHDS-derived patient tumors (Figure 4D). We did not see expression changes associated with genes encoding the mitochondria-associated transcription factors NRF-1 and NRF-2. Taken together, these results indicate that deregulation of FLCN function by point mutation is associated with *FNIP2 *deregulation and perturbation of the PGC-1α-TFAM signaling axis.

### *FLCN *expression inversely correlates with *PGC-1α *activation

**Figure 5:**
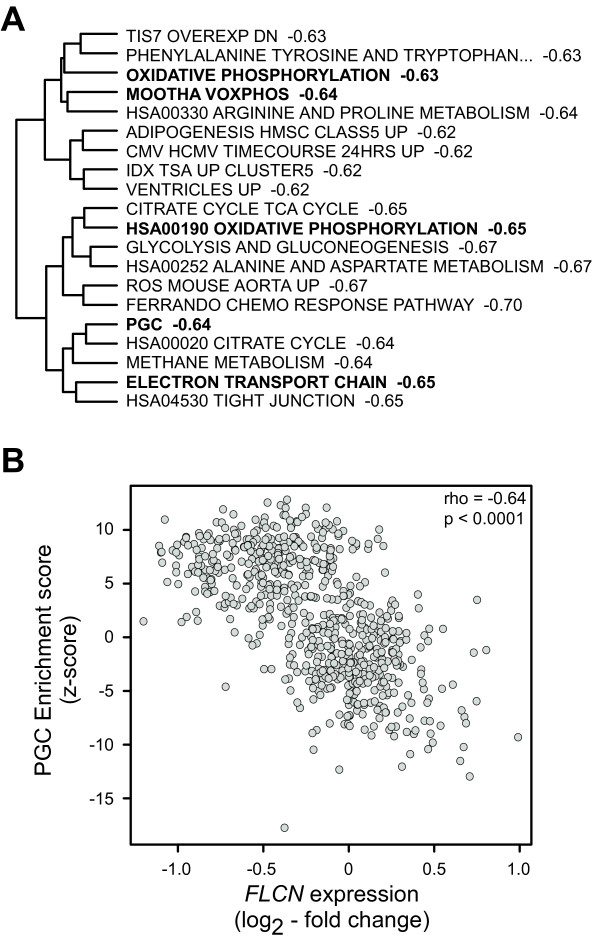
***FLCN *expression negatively correlates with PGC-1α activation**. A) The twenty most highly correlated gene sets with *FLCN *expression levels, followed by their respective Spearman rho correlation coefficients. Bold font indicates gene sets also shown in Figure 3. The dendrogram is based on gene set dissimilarity scores (see Materials and Methods). B) Plot of *FLCN *expression and the enrichment scores for the PGC gene set in tumors of the breast, cervix, colon, kidney, lung, lymph, ovary, pancreas, prostate, stomach, thyroid, and vulva, with tissue type-matched normal tissue. Data for A) and B) are from the Expression Project for Oncology - International Genomics Consortium.

Based on the data from the BHDS-derived tumors, we hypothesized that defects in FLCN may be associated with increased expression of genes related to mitochondria and OXPHOS. To test this hypothesis, we examined the relationship between *FLCN *expression and gene set enrichment in a variety of other tumor tissue types, using a data set that includes tumors of the breast, cervix, colon, kidney, lung, lymph, ovary, pancreas, prostate, stomach, thyroid, and vulva, with matched normal tissue of each tissue type. Using *FLCN *expression levels and PGSEA scores of the 1892 gene sets analyzed previously for this data set, we determined which gene sets were most related to *FLCN *gene expression. Consistent with the loss of FLCN function in BHDS-derived tumors, the top 20 gene sets identified were all negatively correlated to *FLCN *expression and were primarily related to metabolism and mitochondrial function (Figure 5A). Specifically, we found that the PGC gene set and other OXPHOS gene sets were highly negatively correlated with *FLCN *expression across these tumor types (Figure 5B). Though not included in the initial gene set correlation analysis, our PGC-1α over-expression signature (Figure 4C) was also negatively correlated with *FLCN *expression (rho, -0.60, p < 0.0001). Based on our findings, it is likely that a FLCN-PGC-1α-TFAM signaling axis exists and that lack of *FLCN *expression may be an important feature in sporadic tumors of other organs as it is in BHDS-derived renal tumors.

## Discussion

To establish the molecular characteristics of tumors that arise in individuals afflicted with BHDS, we compared gene expression data from renal tumors of BHDS patients with expression data from sporadic renal tumors. Although previous gene expression profiling studies indicated that renal tumors isolated from individuals afflicted with von Hippel-Lindau disease are indistinguishable from sporadic clear cell RCC[43], we show that kidney tumors from patients with BHDS also have unique genetic and cytogenetic characteristics from sporadic renal oncocytoma and chromophobe RCC. In particular, cytogenetic defects that are typical of sporadic oncocytoma and chromophobe RCC, including defects of chromosome 19, loss of chromosome 1, and translocations involving chromosome 11, were largely absent from BHDS-derived tumors. Interestingly, we did not find differences in *FLCN *expression by either our gene expression arrays nor by qRT-PCR, suggesting that the *FLCN *mRNA transcript may not be subject to nonsense-mediated mRNA decay. However, several individual genes are differentially expressed between BHDS-derived tumors and the sporadic tumors. One gene in particular, *DAPL1 *(*death-associated protein-like 1*), is expressed at a high level in BHDS-derived tumors. Although the function of DAPL1 is not known, it was originally termed *early epithelial differentiation associated *(EEDA) for its expression in stratified squamous epithelium, specifically in a population of cells of the hair follicle [60]. High expression of this gene in BHDS-derived tumors is a potentially interesting finding given the clinical presentation of fibrofolliculomas that arise in BHDS-afflicted individuals.

Several recent reports have implicated FLCN in the energy and nutrient signaling pathway through its interactions with FNIP1 and FNIP2 and its indirect interaction with AMPK (Figure 4A). These studies have also suggested that FLCN impacts the mammalian target of rapamycin (mTOR) related components of the PI3K-Akt signal transduction pathway [22]. Consistent with the existence of a FLCN-mTOR relationship, treatment with the specific mTOR inhibitor, rapamycin, delays the death of mice that possess targeted deletion of FLCN in the kidney [61,62]. We noted high expression of *FNIP2 *and *TSC1 *in BHDS-derived tumors, implicating a novel link between FLCN and both AMPK- and mTOR-mediated signaling and transcription. However, we did not see evidence of PI3K-Akt activation in BHDS-derived tumors using an expression signature that was a robust predictor of PI3K-Akt pathway activation in other renal tumors [53], nor did we see consistent enrichment of the three mTOR activation signatures from the MsigDB in the BHDS patient samples. It is possible that the up-regulation of *TSC1 *we have observed represents a feedback effect from the somatic mutation in *FLCN*. One potential rational for this observation is that is has recently been noted that activation of mTOR controls mitochondrial gene expression through signaling with PGC-1α [63]. Moreover, mTOR-mediated control of mitochondrial gene expression is inhibited by application of rapamycin. Our results suggest that the effects of rapamycin noted in FLCN loss-of-function mice may be through the mitochondrial effects of mTOR activation as opposed to activation of PI3K-Akt.

Throughout our analysis, we observed that one sporadic renal oncocytoma co-clustered with the BHDS-derived tumors and showed strong PGC-1α-related gene expression (Figure 1B, F). This tumor sample also lacked the cytogenetic features typical of sporadic oncocytomas, such as loss of chromosome 1, deregulation of *CCND1*, and over-expression of chromosome 19 genes (Figure 2). Interestingly, this individual presented with renal oncocytoma at the age of 34 years old, while the median age of sporadic renal oncocytoma is between 65-70 [7,64]. Given that early age at diagnosis (under age 50) is often a feature of hereditary disease, we sequenced the entire *FLCN *open reading frame from non-diseased kidney tissue of this patient and only identified a common single nucleotide polymorphism within the 5' UTR[11]. Though somatic mutations in *FLCN *occur in approximately 10 percent of sporadic tumors, we lacked the tissue required to determine the *FLCN *status in the tumor itself. However, these results suggest that a separate BHDS-like group of sporadic renal oncocytomas could exist in the population, genetically distinct from other sporadic renal tumors.

Finally, although these DCT-derived tumors are genetically distinct, BHDS-derived tumors, sporadic renal oncocytoma, and chromophobe RCC share their histological and mitochondrial/OXHPOS gene expression characteristics. Development of oncocytomas in organ sites outside of the kidney are also associated with prominent mitochondrial DNA mutations, a high production of mitochondria, and deregulated OXPHOS gene expression [65,66]. In renal oncocytoma and other mitochondrial myopathies, up-regulation of mitochondrial gene expression is thought to represent a feedback mechanism to compensate for mitochondrial damage [67,68]. In this study, we show that the mitochondrial expression phenotype is even more pronounced in samples that harbor *FLCN *mutations. The enhanced mitochondrial gene expression in BHDS samples suggests that wild-type *FLCN *is important for efficient mitochondrial function and that lack of functional FLCN leads to a yet unknown mitochondrial dysfunction. Deregulation of mitochondrial proteins has recently been identified in sporadic oncocytoma and chromophobe RCC [30]. Future studies will therefore help to clarify the role of FLCN in mitochondrial function.

## Conclusions

Our results support a genetic distinction between BHDS-associated tumors and other sporadic renal neoplasias. In addition, we found that deregulation of the PGC-1α-TFAM signaling axis is most pronounced in renal tumors that harbor *FLCN *mutations and in tumors from other organs that have relatively low expression of *FLCN*. These results are consistent with the recently discovered interaction between FLCN and AMPK and support a model in which FLCN is a regulator of mitochondrial function.

## Authors' contributions

DP generated the gene expression data, while JK performed the data analysis with datasets obtained from KD. JK, DP, JC, NN and JM carried out the molecular studies. JK, XY, AS, PZ, MA, MN, UB, SG, SG, YD, LY, AM, VV, SR and BT participated in the study design, mutation detection, pathological evaluation, and sample collection. JK and KF drafted the manuscript. All authors read and approved the final manuscript.

## Competing interests

The authors declare that they have no competing interests.

## Pre-publication history

The pre-publication history for this paper can be accessed here:

http://www.biomedcentral.com/1755-8794/3/59/prepub

## Supplementary Material

Additional file 1 **Supplementary Tables S1-S4**. This file contains four supplementary tables: Table S1- characteristics of BHD-derived tumor samples, Table S2- top 200 genes differentially expressed between BHD renal tumors and sporadic renal oncocytomas (ON) and chromophobe RCC (CH) samples, Table S3- most significantly enriched gene sets in BHDS-derived tumor samples versus sporadic oncocytoma (ON) and chromophobe RCC (CH) samples, and Table S4- primer and probe sequences for qRT-PCR validation of genes in BHDS, CH, ON, and CC tumors relative to Normal kidney. Click here for file

Additional file 2 **Supplementary Figures S1-S3**. This file contains three supplementary figures: Figure S1- gene expression measurements for individual genes deregulated in BHDS tumors, Figure S2- heatmap of differentially expressed genes from Figure 1E in sporadic kidney tumors, and Figure S3- histological images of sample BHD4. Click here for file
