# Supplementary material for: Birt-Hogg-Dubé renal tumors are genetically distinct from other renal neoplasias and are associated with up-regulation of mitochondrial gene expression
Source: BMC Med Genomics. 2010 Dec 16;3:59. doi: 10.1186/1755-8794-3-59 (PMC3012009; doi:10.1186/1755-8794-3-59)
Supplement: Additional file 1 — Supplementary Tables S1-S4. This file contains four supplementary tables: Table S1- characteristics of BHD-derived tumor samples, Table S2- top 200 genes differentially expressed between BHD renal tumors and sporadic renal oncocytomas (ON) and chromophobe RCC (CH) samples, Table S3- most significantly enriched gene sets in BHDS-derived tumor samples versus sporadic oncocytoma (ON) and chromophobe RCC (CH) samples, and Table S4- primer and probe sequences for qRT-PCR validation of genes in BHDS, CH, ON, and CC tumors relative to Normal kidney. [file 1755-8794-3-59-S1.DOC]

**Table S1. Characteristics of BHD-derived tumor samples**

| **Patient** | **Sex** | **Germline FLCN mutation** | **Protein annotation** | **Type** | **Sample** | **Histology** | **Date expression profiling performed** |
| --- | --- | --- | --- | --- | --- | --- | --- |
| BHD1 | M | c.779+1G>T | - | splice site | DT017^a,1^ | Chromophobe | 12/29/2004; 02/21/2008 |
|  |  |  |  |  | DT004^b,1^ | Chromophobe | 02/21/2008 |
| BHD2 | F | c.375T>A | p.Cys125X | nonsense | NW14^2^ | Hybrid | 03/25/2008 |
| BHD3 | M | c.1062+2T>G | - | splice site | APO3 6272^3^ | Hybrid | 10/23/2009 |
| BHD4 | M | c.1285delC | p.His429ThrfsX39 | frameshift | P07 1839^4^ | Hybrid | 10/23/2009 |

^a^Duplicate arrays performed on this tumor.

^b^Second tumor removed from patient approximately one year later.

Samples collected from ^1^Karonlinska University Hospital, Stockholm, Sweden, ^2^Northwestern University, Chicago, IL, USA, ^3^Necker University Hospital, Paris, France, and ^4^Foch Hospital, Suresnes, France.

Table S2. Top 200 genes differentially expressed between BHD renal tumors and sporadic renal oncocytomas (ON) and chromophobe RCC (CH) samples

| **Entrez Gene ID** | **Symbol** | **Description** | **Fold-change (ON)** | **Adj. p-value (ON)** | **Fold-change (CH)** | **Adj. p-value (CH)** |
| --- | --- | --- | --- | --- | --- | --- |
| 57467 | HHATL | hedgehog acyltransferase-like | 5.95 | 2.54E-04 | 4.80 | 3.15E-03 |
| 92196 | DAPL1 | death associated protein-like 1 | 4.00 | 9.76E-03 | 4.49 | 1.96E-04 |
| 145741 | FAM148A | family with sequence similarity 148, member A | 4.88 | 1.55E-07 | 4.89 | 1.17E-08 |
| 6750 | SST | somatostatin | 4.28 | 6.46E-06 | 3.36 | 1.18E-03 |
| 28513 | CDH19 | cadherin 19, type 2 | 3.53 | 1.84E-03 | 4.05 | 9.27E-07 |
| 54504 | CPVL | carboxypeptidase, vitellogenic-like | 3.86 | 8.53E-06 | 3.44 | 8.16E-05 |
| 10875 | FGL2 | fibrinogen-like 2 | 3.57 | 1.80E-04 | 4.00 | 6.77E-09 |
| 3880 | KRT19 | keratin 19 | 3.19 | 1.31E-03 | 3.71 | 1.16E-06 |
| 7103 | TSPAN8 | tetraspanin 8 | 3.39 | 1.80E-04 | 3.65 | 1.97E-07 |
| 730124 | NA | NA | 3.63 | 5.03E-05 | 3.29 | 6.27E-08 |
| 4232 | MEST | mesoderm specific transcript homolog (mouse) | 2.98 | 1.26E-03 | 3.49 | 6.77E-09 |
| 3758 | KCNJ1 | potassium inwardly-rectifying channel, subfamily J, member 1 | 3.40 | 1.68E-04 | 3.43 | 3.31E-05 |
| 3773 | KCNJ16 | potassium inwardly-rectifying channel, subfamily J, member 16 | 3.07 | 4.35E-04 | 3.40 | 4.64E-07 |
| 9034 | CCRL2 | chemokine (C-C motif) receptor-like 2 | 3.26 | 4.35E-04 | 2.84 | 1.49E-04 |
| 6557 | SLC12A1 | solute carrier family 12 (sodium/potassium/chloride transporters), member 1 | 3.23 | 3.50E-04 | 2.91 | 3.05E-03 |
| 9122 | SLC16A4 | solute carrier family 16, member 4 (monocarboxylic acid transporter 5) | 2.11 | 5.65E-03 | 3.28 | 1.18E-06 |
| 80059 | LRRTM4 | leucine rich repeat transmembrane neuronal 4 | 2.94 | 4.28E-03 | 2.96 | 1.21E-03 |
| 3426 | CFI | complement factor I | 2.41 | 2.33E-03 | 2.84 | 1.16E-05 |
| 53346 | TM6SF1 | transmembrane 6 superfamily member 1 | 2.82 | 1.73E-03 | 2.74 | 5.00E-06 |
| 825 | CAPN3 | calpain 3, (p94) | 2.56 | 4.53E-03 | 2.81 | 1.14E-06 |
| 54997 | TESC | tescalcin | 2.48 | 4.71E-04 | 2.73 | 6.23E-07 |
| 27233 | SULT1C4 | sulfotransferase family, cytosolic, 1C, member 4 | 2.57 | 2.45E-04 | 2.93 | 1.01E-05 |
| 4129 | MAOB | monoamine oxidase B | 2.10 | 2.58E-03 | 2.57 | 1.65E-04 |
| 9258 | MFHAS1 | malignant fibrous histiocytoma amplified sequence 1 | 2.21 | 3.85E-03 | 2.76 | 1.05E-05 |
| 8601 | RGS20 | regulator of G-protein signaling 20 | 2.66 | 5.61E-06 | 2.64 | 1.92E-07 |
| 8972 | MGAM | maltase-glucoamylase (alpha-glucosidase) | 2.84 | 8.53E-06 | 2.24 | 2.52E-04 |
| 25878 | MXRA5 | matrix-remodelling associated 5 | 2.46 | 7.28E-04 | 2.53 | 2.21E-05 |
| 80704 | SLC19A3 | solute carrier family 19, member 3 | 2.41 | 9.56E-05 | 2.62 | 1.63E-06 |
| 8406 | SRPX | sushi-repeat-containing protein, X-linked | 2.39 | 3.42E-04 | 2.51 | 1.46E-03 |
| 84419 | C15orf48 | chromosome 15 open reading frame 48 | 2.64 | 8.80E-04 | 2.46 | 2.45E-04 |
| 347733 | TUBB2B | tubulin, beta 2B | 2.99 | 5.76E-04 | 2.00 | 7.43E-03 |
| 55 | ACPP | acid phosphatase, prostate | 2.42 | 1.17E-03 | 2.30 | 3.34E-04 |
| 2770 | GNAI1 | guanine nucleotide binding protein (G protein), alpha inhibiting activity polypeptide 1 | 2.68 | 5.11E-06 | 1.74 | 9.82E-04 |
| 9365 | KL | klotho | 1.92 | 2.21E-03 | 2.50 | 3.93E-04 |
| 6446 | SGK1 | serum/glucocorticoid regulated kinase 1 | 2.64 | 2.11E-04 | 2.24 | 6.71E-04 |
| 127495 | LRRC39 | leucine rich repeat containing 39 | 2.03 | 1.27E-03 | 2.24 | 1.59E-05 |
| 26353 | HSPB8 | heat shock 22kDa protein 8 | 2.76 | 2.11E-04 | 1.74 | 6.36E-03 |
| 64332 | NFKBIZ | nuclear factor of kappa light polypeptide gene enhancer in B-cells inhibitor, zeta | 2.47 | 3.06E-03 | 2.05 | 2.27E-03 |
| 23150 | FRMD4B | FERM domain containing 4B | 2.20 | 4.70E-03 | 2.15 | 1.26E-03 |
| 6662 | SOX9 | SRY (sex determining region Y)-box 9 | 2.65 | 4.90E-04 | 2.04 | 2.75E-03 |
| 90293 | KLHL13 | kelch-like 13 (Drosophila) | 2.18 | 3.82E-03 | 1.93 | 1.72E-03 |
| 8671 | SLC4A4 | solute carrier family 4, sodium bicarbonate cotransporter, member 4 | 2.18 | 1.44E-06 | 2.12 | 2.45E-06 |
| 22996 | TTC39A | tetratricopeptide repeat domain 39A | 1.79 | 1.64E-03 | 2.32 | 5.95E-06 |
| 160335 | TMTC2 | transmembrane and tetratricopeptide repeat containing 2 | 2.20 | 7.72E-04 | 2.01 | 5.11E-04 |
| 23414 | ZFPM2 | zinc finger protein, multitype 2 | 1.73 | 2.56E-03 | 2.24 | 2.37E-07 |
| 5095 | PCCA | propionyl Coenzyme A carboxylase, alpha polypeptide | 1.50 | 4.03E-03 | 2.60 | 2.79E-04 |
| 55586 | MIOX | myo-inositol oxygenase | 1.91 | 7.01E-03 | 2.17 | 6.77E-04 |
| 30812 | SOX8 | SRY (sex determining region Y)-box 8 | 1.51 | 8.41E-03 | 2.32 | 3.89E-04 |
| 2262 | GPC5 | glypican 5 | 2.11 | 2.56E-03 | 2.09 | 6.89E-04 |
| 353189 | SLCO4C1 | solute carrier organic anion transporter family, member 4C1 | 2.04 | 4.03E-03 | 1.86 | 1.57E-03 |
| 8910 | SGCE | sarcoglycan, epsilon | 2.25 | 3.25E-03 | 1.98 | 9.15E-03 |
| 7164 | TPD52L1 | tumor protein D52-like 1 | 1.73 | 8.19E-05 | 2.43 | 1.95E-06 |
| 79623 | GALNT14 | UDP-N-acetyl-alpha-D-galactosamine:polypeptide N-acetylgalactosaminyltransferase 14 (GalNAc-T14) | 1.78 | 1.48E-03 | 2.00 | 2.68E-05 |
| 29982 | NRBF2 | nuclear receptor binding factor 2 | 1.63 | 9.92E-03 | 1.90 | 2.11E-04 |
| 5101 | PCDH9 | protocadherin 9 | 1.91 | 1.03E-03 | 1.79 | 3.14E-04 |
| 64922 | LRRC19 | leucine rich repeat containing 19 | 1.66 | 5.81E-03 | 1.96 | 4.28E-05 |
| 54769 | DIRAS2 | DIRAS family, GTP-binding RAS-like 2 | 1.97 | 1.44E-03 | 1.99 | 2.47E-04 |
| 6355 | CCL8 | chemokine (C-C motif) ligand 8 | 1.86 | 6.33E-03 | 1.79 | 5.15E-03 |
| 7280 | TUBB2A | tubulin, beta 2A | 2.21 | 1.03E-03 | 1.70 | 1.11E-03 |
| 25975 | EGFL6 | EGF-like-domain, multiple 6 | 1.93 | 1.59E-04 | 1.95 | 2.21E-05 |
| 8739 | HRK | harakiri, BCL2 interacting protein (contains only BH3 domain) | 2.12 | 1.34E-03 | 1.61 | 3.48E-03 |
| 7358 | UGDH | UDP-glucose dehydrogenase | 1.76 | 3.17E-04 | 2.06 | 1.21E-05 |
| 10486 | CAP2 | CAP, adenylate cyclase-associated protein, 2 (yeast) | 1.80 | 3.99E-05 | 2.06 | 1.97E-07 |
| 6296 | ACSM3 | acyl-CoA synthetase medium-chain family member 3 | 1.50 | 9.22E-03 | 1.84 | 6.81E-04 |
| 221749 | C6orf145 | chromosome 6 open reading frame 145 | 1.67 | 1.08E-03 | 1.98 | 6.97E-06 |
| 1524 | CX3CR1 | chemokine (C-X3-C motif) receptor 1 | 2.01 | 3.50E-03 | 1.54 | 7.16E-03 |
| 54510 | PCDH18 | protocadherin 18 | 1.82 | 6.36E-04 | 1.76 | 2.27E-04 |
| 23603 | CORO1C | coronin, actin binding protein, 1C | 1.60 | 8.86E-03 | 1.63 | 2.68E-04 |
| 26002 | MOXD1 | monooxygenase, DBH-like 1 | 1.68 | 1.82E-03 | 1.76 | 3.07E-05 |
| 1687 | DFNA5 | deafness, autosomal dominant 5 | 1.43 | 2.40E-03 | 2.05 | 9.95E-07 |
| 55733 | HHAT | hedgehog acyltransferase | 1.86 | 1.14E-03 | 1.91 | 1.12E-05 |
| 3434 | IFIT1 | interferon-induced protein with tetratricopeptide repeats 1 | 1.47 | 1.62E-03 | 1.80 | 9.81E-06 |
| 7357 | UGCG | UDP-glucose ceramide glucosyltransferase | 2.26 | 2.85E-03 | 1.71 | 7.79E-03 |
| 427 | ASAH1 | N-acylsphingosine amidohydrolase (acid ceramidase) 1 | 1.68 | 1.62E-03 | 1.68 | 2.62E-04 |
| 2947 | GSTM3 | glutathione S-transferase mu 3 (brain) | 1.66 | 4.44E-04 | 1.75 | 1.64E-04 |
| 9071 | CLDN10 | claudin 10 | 1.62 | 3.45E-04 | 1.73 | 5.47E-06 |
| 873 | CBR1 | carbonyl reductase 1 | 1.80 | 1.28E-03 | 1.91 | 2.00E-05 |
| 929 | CD14 | CD14 molecule | 2.02 | 8.42E-05 | 1.33 | 3.24E-03 |
| 2948 | GSTM4 | glutathione S-transferase mu 4 | 1.71 | 2.45E-04 | 1.80 | 2.68E-05 |
| 65124 | ANKRD57 | ankyrin repeat domain 57 | 1.49 | 2.99E-03 | 1.56 | 6.49E-04 |
| 8925 | HERC1 | hect (homologous to the E6-AP (UBE3A) carboxyl terminus) domain and RCC1 (CHC1)-like domain (RLD) 1 | 1.61 | 2.26E-04 | 1.66 | 5.01E-05 |
| 26235 | FBXL4 | F-box and leucine-rich repeat protein 4 | 1.39 | 9.15E-04 | 1.75 | 5.63E-05 |
| 6519 | SLC3A1 | solute carrier family 3 (cystine, dibasic and neutral amino acid transporters, activator of cystine, dibasic and neutral amino acid transport), member 1 | 0.90 | 7.77E-03 | 3.05 | 1.68E-03 |
| 29108 | PYCARD | PYD and CARD domain containing | 1.78 | 1.97E-03 | 1.43 | 2.18E-03 |
| 192669 | EIF2C3 | eukaryotic translation initiation factor 2C, 3 | 1.67 | 1.26E-03 | 1.70 | 3.62E-04 |
| 57179 | KIAA1191 | KIAA1191 | 1.67 | 6.72E-04 | 1.60 | 1.12E-04 |
| 22977 | AKR7A3 | aldo-keto reductase family 7, member A3 (aflatoxin aldehyde reductase) | 1.68 | 4.08E-03 | 1.60 | 3.51E-04 |
| 341640 | FREM2 | FRAS1 related extracellular matrix protein 2 | 1.63 | 1.51E-03 | 1.51 | 1.24E-03 |
| 4747 | NEFL | neurofilament, light polypeptide | 1.43 | 5.35E-03 | 1.66 | 7.88E-05 |
| 30011 | SH3KBP1 | SH3-domain kinase binding protein 1 | 1.50 | 6.36E-04 | 1.38 | 4.90E-04 |
| 10579 | TACC2 | transforming, acidic coiled-coil containing protein 2 | 1.41 | 1.50E-03 | 1.42 | 1.34E-04 |
| 220213 | OTUD1 | OTU domain containing 1 | 1.51 | 2.17E-03 | 1.61 | 2.44E-04 |
| 10129 | FRY | furry homolog (Drosophila) | 1.51 | 2.07E-03 | 1.52 | 3.80E-04 |
| 283537 | SLC46A3 | solute carrier family 46, member 3 | 1.10 | 8.76E-03 | 1.92 | 2.83E-04 |
| 443 | ASPA | aspartoacylase (Canavan disease) | 1.44 | 1.28E-03 | 1.42 | 1.37E-03 |
| 9971 | NR1H4 | nuclear receptor subfamily 1, group H, member 4 | 1.46 | 5.44E-04 | 1.40 | 2.88E-04 |
| 134285 | TMEM171 | transmembrane protein 171 | 1.30 | 6.63E-03 | 1.85 | 4.75E-03 |
| 51491 | NOP16 | NOP16 nucleolar protein homolog (yeast) | 1.48 | 4.58E-03 | 1.49 | 8.14E-05 |
| 57188 | ADAMTSL3 | ADAMTS-like 3 | 1.35 | 5.47E-03 | 1.45 | 2.31E-04 |
| 145389 | SLC38A6 | solute carrier family 38, member 6 | 1.36 | 1.68E-03 | 1.40 | 4.37E-04 |
| 2213 | FCGR2B | Fc fragment of IgG, low affinity IIb, receptor (CD32) | -1.15 | 5.33E-03 | -1.02 | 1.84E-03 |
| 90835 | C16orf93 | chromosome 16 open reading frame 93 | -1.15 | 3.33E-03 | -1.15 | 6.69E-04 |
| 129684 | CNTNAP5 | contactin associated protein-like 5 | -1.29 | 3.85E-03 | -1.12 | 3.01E-04 |
| 100128309 | NA | NA | -1.07 | 2.21E-03 | -1.54 | 2.92E-04 |
| 51200 | CPA4 | carboxypeptidase A4 | -1.17 | 8.54E-03 | -1.16 | 4.04E-04 |
| 2980 | GUCA2A | guanylate cyclase activator 2A (guanylin) | -1.05 | 5.84E-03 | -1.07 | 4.59E-04 |
| 729642 | NA | NA | -1.16 | 2.78E-03 | -1.02 | 5.30E-04 |
| 8125 | ANP32A | acidic (leucine-rich) nuclear phosphoprotein 32 family, member A | -1.16 | 2.85E-03 | -1.1 | 5.17E-04 |
| 9768 | KIAA0101 | KIAA0101 | -1.28 | 1.51E-03 | -1.01 | 2.00E-04 |
| 6649 | SOD3 | superoxide dismutase 3, extracellular | -1.18 | 3.46E-03 | -1.11 | 6.61E-04 |
| 4142 | MAS1 | MAS1 oncogene | -1.11 | 4.50E-03 | -1.11 | 1.27E-03 |
| 10046 | MAMLD1 | mastermind-like domain containing 1 | -1.15 | 1.39E-03 | -1.05 | 1.92E-03 |
| 2842 | GPR19 | G protein-coupled receptor 19 | -1.07 | 2.74E-03 | -1.03 | 9.10E-04 |
| 84070 | FAM186B | family with sequence similarity 186, member B | -1.06 | 1.25E-03 | -1.01 | 7.89E-05 |
| 283314 | UNQ2963 | hypothetical LOC283314 | -1.02 | 2.70E-03 | -1.07 | 4.55E-04 |
| 387707 | CC2D2B | coiled-coil and C2 domain containing 2B | -1.09 | 2.64E-03 | -1.11 | 1.01E-03 |
| 2550 | GABBR1 | gamma-aminobutyric acid (GABA) B receptor, 1 | -1.22 | 2.12E-03 | -1.03 | 2.44E-03 |
| 117156 | SCGB3A2 | secretoglobin, family 3A, member 2 | -1.06 | 1.37E-03 | -1.01 | 4.81E-04 |
| 255926 | ADAM5P | ADAM metallopeptidase domain 5 pseudogene | -1.16 | 5.23E-03 | -1.06 | 1.08E-03 |
| 9746 | CLSTN3 | calsyntenin 3 | -1.13 | 6.27E-03 | -1.17 | 6.76E-04 |
| 403314 | APOBEC4 | apolipoprotein B mRNA editing enzyme, catalytic polypeptide-like 4 (putative) | -1.04 | 2.39E-03 | -1.06 | 2.20E-04 |
| 84103 | C4orf17 | chromosome 4 open reading frame 17 | -1.13 | 3.17E-03 | -1.05 | 9.85E-04 |
| 116369 | SLC26A8 | solute carrier family 26, member 8 | -1.32 | 7.07E-03 | -1.34 | 5.40E-04 |
| 147138 | TMC8 | transmembrane channel-like 8 | -1.22 | 3.09E-03 | -1.15 | 1.65E-03 |
| 10266 | RAMP2 | receptor (G protein-coupled) activity modifying protein 2 | -1.17 | 1.14E-03 | -1.03 | 2.20E-03 |
| 57335 | ZNF286A | zinc finger protein 286A | -1.47 | 1.79E-03 | -1.39 | 1.31E-03 |
| 79908 | BTNL8 | butyrophilin-like 8 | -1.27 | 1.86E-03 | -1.26 | 4.61E-04 |
| 727961 | hCG_1776047 | hCG1776047 | -1.3 | 1.79E-03 | -1.25 | 8.34E-04 |
| 51564 | HDAC7 | histone deacetylase 7 | -1.09 | 6.95E-03 | -1.22 | 1.51E-04 |
| 79727 | LIN28 | lin-28 homolog (C. elegans) | -1.3 | 3.82E-03 | -1.28 | 4.04E-04 |
| 57592 | ZNF687 | zinc finger protein 687 | -1.04 | 2.55E-03 | -1 | 5.89E-04 |
| 4059 | BCAM | basal cell adhesion molecule (Lutheran blood group) | -1.05 | 2.24E-03 | -1.51 | 1.95E-04 |
| 4825 | NKX6-1 | NK6 homeobox 1 | -1.06 | 2.70E-03 | -1.67 | 2.59E-04 |
| 29968 | PSAT1 | phosphoserine aminotransferase 1 | -1.48 | 3.78E-03 | -1.27 | 2.09E-04 |
| 4632 | MYL1 | myosin, light chain 1, alkali; skeletal, fast | -1.22 | 2.62E-03 | -1.27 | 2.60E-04 |
| 114769 | CARD16 | caspase recruitment domain family, member 16 | -1.22 | 1.26E-03 | -1.23 | 5.63E-04 |
| 5866 | RAB3IL1 | RAB3A interacting protein (rabin3)-like 1 | -1.11 | 7.45E-04 | -1.12 | 8.56E-05 |
| 9123 | SLC16A3 | solute carrier family 16, member 3 (monocarboxylic acid transporter 4) | -1.32 | 2.89E-03 | -1.28 | 9.58E-03 |
| 6947 | TCN1 | transcobalamin I (vitamin B12 binding protein, R binder family) | -1.1 | 2.49E-03 | -1.1 | 5.57E-04 |
| 729994 | LOC729994 | hypothetical LOC729994 | -1.37 | 1.98E-03 | -1.08 | 3.72E-03 |
| 3779 | KCNMB1 | potassium large conductance calcium-activated channel, subfamily M, beta member 1 | -1.13 | 3.91E-03 | -1.11 | 1.40E-03 |
| 646482 | LOC646482 | hypothetical LOC646482 | -1.48 | 1.85E-03 | -1.32 | 7.92E-04 |
| 5271 | SERPINB8 | serpin peptidase inhibitor, clade B (ovalbumin), member 8 | -1.27 | 1.64E-03 | -1.11 | 9.86E-04 |
| 78991 | PCYOX1L | prenylcysteine oxidase 1 like | -1.1 | 1.79E-03 | -1.26 | 2.02E-04 |
| 10903 | MTMR11 | myotubularin related protein 11 | -1.24 | 1.14E-03 | -1.16 | 2.73E-04 |
| 3787 | KCNS1 | potassium voltage-gated channel, delayed-rectifier, subfamily S, member 1 | -1.18 | 1.01E-03 | -1.2 | 2.18E-04 |
| 5063 | PAK3 | p21 protein (Cdc42/Rac)-activated kinase 3 | -1.32 | 2.88E-03 | -1.28 | 9.61E-04 |
| 183 | AGT | angiotensinogen (serpin peptidase inhibitor, clade A, member 8) | -1.15 | 8.12E-04 | -1.14 | 2.24E-04 |
| 64232 | MS4A5 | membrane-spanning 4-domains, subfamily A, member 5 | -1.18 | 3.09E-03 | -1.08 | 1.76E-03 |
| 441642 | RPL7AP9 | ribosomal protein L7a pseudogene 9 | -1.19 | 1.48E-03 | -1.31 | 8.21E-05 |
| 83755 | KRTAP4-12 | keratin associated protein 4-12 | -1.21 | 4.44E-04 | -1.1 | 1.06E-03 |
| 28815 | IGLV2-14 | immunoglobulin lambda variable 2-14 | -1.14 | 4.19E-03 | -1.23 | 3.22E-04 |
| 79741 | C10orf68 | chromosome 10 open reading frame 68 | -1.2 | 1.25E-03 | -1.1 | 6.99E-04 |
| 80350 | LPAL2 | lipoprotein, Lp(a)-like 2 pseudogene | -1.24 | 2.12E-03 | -1.25 | 1.44E-04 |
| 9293 | GPR52 | G protein-coupled receptor 52 | -1.18 | 1.45E-03 | -1.16 | 5.17E-04 |
| 100128126 | LOC100128126 | hypothetical protein LOC100128126 | -1.35 | 1.28E-03 | -1.1 | 8.06E-04 |
| 10214 | SSX3 | synovial sarcoma, X breakpoint 3 | -1.31 | 3.12E-03 | -1.23 | 8.75E-04 |
| 1579 | CYP4A11 | cytochrome P450, family 4, subfamily A, polypeptide 11 | -1.22 | 7.83E-03 | -1.21 | 3.07E-03 |
| 10050 | SLC17A4 | solute carrier family 17 (sodium phosphate), member 4 | -1.26 | 2.33E-03 | -1.29 | 2.18E-04 |
| 196410 | METTL7B | methyltransferase like 7B | -1.2 | 1.48E-03 | -1.02 | 3.80E-04 |
| 85291 | KRTAP4-2 | keratin associated protein 4-2 | -1.4 | 1.28E-03 | -1.25 | 4.99E-04 |
| 220979 | C10orf25 | chromosome 10 open reading frame 25 | -1.28 | 2.59E-03 | -1.3 | 2.48E-04 |
| 142689 | ASB12 | ankyrin repeat and SOCS box-containing 12 | -1.4 | 1.01E-03 | -1.47 | 1.98E-04 |
| 57529 | RGAG1 | retrotransposon gag domain containing 1 | -1.34 | 1.28E-03 | -1.34 | 2.62E-04 |
| 51617 | HMP19 | HMP19 protein | -1.24 | 3.09E-03 | -1.28 | 1.84E-04 |
| 282616 | IL28A | interleukin 28A (interferon, lambda 2) | -1.29 | 8.36E-04 | -1.19 | 3.64E-04 |
| 147968 | CAPN12 | calpain 12 | -1.11 | 1.48E-03 | -1.43 | 4.07E-05 |
| 91544 | UBXN11 | UBX domain protein 11 | -1.31 | 7.66E-04 | -1.27 | 1.07E-04 |
| 117852 | TRIM78P | tripartite motif-containing 78, pseudogene | -1.54 | 3.76E-03 | -1.46 | 1.16E-03 |
| 26776 | SNORA71B | small nucleolar RNA, H/ACA box 71B | -1.58 | 1.14E-03 | -1.54 | 2.45E-04 |
| 3804 | KIR2DL3 | killer cell immunoglobulin-like receptor, two domains, long cytoplasmic tail, 3 | -1.28 | 4.19E-03 | -1.26 | 1.70E-03 |
| 57571 | ATPGD1 | ATP-grasp domain containing 1 | -1.52 | 3.17E-04 | -1.2 | 4.18E-04 |
| 5670 | PSG2 | pregnancy specific beta-1-glycoprotein 2 | -1.43 | 1.88E-03 | -1.29 | 1.42E-03 |
| 92346 | C1orf105 | chromosome 1 open reading frame 105 | -1.45 | 7.66E-04 | -1.41 | 2.23E-04 |
| 140683 | C20orf70 | chromosome 20 open reading frame 70 | -1.34 | 4.56E-03 | -1.64 | 4.59E-05 |
| 51267 | CLEC1A | C-type lectin domain family 1, member A | -1.57 | 1.86E-03 | -1.48 | 8.45E-04 |
| 126393 | HSPB6 | heat shock protein, alpha-crystallin-related, B6 | -1.59 | 1.39E-03 | -1.55 | 6.81E-04 |
| 147646 | LOC147646 | hypothetical protein LOC147646 | -1.38 | 7.70E-04 | -1.45 | 1.42E-04 |
| 284358 | MAMSTR | MEF2 activating motif and SAP domain containing transcriptional regulator | -1.47 | 4.32E-03 | -1.53 | 4.91E-04 |
| 2065 | ERBB3 | v-erb-b2 erythroblastic leukemia viral oncogene homolog 3 (avian) | -1.99 | 6.72E-04 | -1.53 | 9.08E-04 |
| 732253 | TDRG1 | testis development related protein 1 | -1.65 | 1.63E-03 | -1.6 | 4.19E-04 |
| 203562 | TMEM31 | transmembrane protein 31 | -1.47 | 1.68E-03 | -1.51 | 7.88E-05 |
| 126364 | LRRC25 | leucine rich repeat containing 25 | -1.43 | 5.76E-04 | -1.46 | 1.51E-04 |
| 6370 | CCL25 | chemokine (C-C motif) ligand 25 | -1.52 | 1.63E-03 | -1.52 | 3.91E-04 |
| 100127980 | LOC100127980 | hypothetical protein LOC100127980 | -1.75 | 4.28E-03 | -1.65 | 1.04E-03 |
| 1559 | CYP2C9 | cytochrome P450, family 2, subfamily C, polypeptide 9 | -1.61 | 6.34E-04 | -1.58 | 2.16E-04 |
| 342897 | NCCRP1 | non-specific cytotoxic cell receptor protein 1 homolog (zebrafish) | -1.8 | 7.66E-04 | -1.69 | 1.84E-04 |
| 138474 | TAF1L | TAF1 RNA polymerase II, TATA box binding protein (TBP)-associated factor, 210kDa-like | -1.69 | 8.47E-04 | -1.74 | 2.21E-04 |
| 5646 | PRSS3 | protease, serine, 3 | -1.73 | 1.62E-03 | -1.72 | 2.21E-04 |
| 114836 | SLAMF6 | SLAM family member 6 | -1.81 | 8.26E-04 | -1.73 | 2.88E-04 |
| 9369 | NRXN3 | neurexin 3 | -1.33 | 6.47E-03 | -3.08 | 3.75E-05 |
| 54065 | FAM165B | family with sequence similarity 165, member B | -2 | 9.25E-04 | -2.17 | 2.70E-05 |
| 389084 | C2orf82 | chromosome 2 open reading frame 82 | -1.93 | 9.56E-05 | -2.05 | 5.95E-06 |
| 286183 | NKAIN3 | Na+/K+ transporting ATPase interacting 3 | -1.82 | 6.99E-04 | -1.87 | 6.16E-05 |
| 90827 | ZNF479 | zinc finger protein 479 | -2.14 | 9.29E-03 | -1.9 | 2.32E-03 |
| 441212 | RP9P | retinitis pigmentosa 9 pseudogene | -1.9 | 1.80E-04 | -1.91 | 4.64E-05 |
| 144124 | OR10A5 | olfactory receptor, family 10, subfamily A, member 5 | -2.24 | 8.26E-04 | -2.22 | 1.54E-04 |
| 978 | CDA | cytidine deaminase | -2.63 | 2.88E-04 | -2.54 | 3.41E-06 |
| 283694 | OR4N4 | olfactory receptor, family 4, subfamily N, member 4 | -2.14 | 8.38E-03 | -2.19 | 2.97E-03 |
| 284422 | C19orf77 | chromosome 19 open reading frame 77 | -3.17 | 9.56E-05 | -3.85 | 1.45E-07 |

Genes that are differentially expressed in BHDS-derived tumors as compared to sporadic renal oncocytomas (FDR<0.01) and chromophobe RCC tumors (FDR<0.01). The genes are ordered based on differences in average expression in BHDS-derived tumors relative to the median expression in the sporadic renal oncocytoma and chromophobe RCC tumors.

**Table S3. Most significantly enriched gene sets in BHDS-derived tumor samples versus sporadic oncocytoma (ON) and chromophobe RCC (CH) samples**

| **Gene Sets** | **Fold-change (ON)** | **Adj. p-value (ON)** | **Fold-change (CH)** | **Adj. p-value (ON)** |
| --- | --- | --- | --- | --- |
| ELECTRON_TRANSPORT_CHAIN | 4.43 | 8.57E-03 | 7.00 | 4.65E-06 |
| MOOTHA_VOXPHOS | 4.18 | 8.30E-03 | 6.73 | 4.65E-06 |
| MITOCHONDRIA | 5.71 | 1.53E-02 | 9.65 | 4.65E-06 |
| HUMAN_MITODB_6_2002 | 6.05 | 1.28E-02 | 10.32 | 3.37E-06 |
| PGC | 5.91 | 5.80E-03 | 7.22 | 4.92E-06 |
| AGEING_KIDNEY_DN | 2.81 | 3.45E-02 | 3.79 | 2.92E-04 |
| AGEING_KIDNEY_SPECIFIC_DN | 3.53 | 3.29E-02 | 4.90 | 2.75E-04 |
| UBIQUINONE_BIOSYNTHESIS | 1.71 | 2.88E-02 | 3.16 | 2.12E-04 |
| STEMCELL_COMMON_UP | 4.24 | 3.31E-02 | 6.99 | 1.02E-04 |
| UVC_XPCS_8HR_DN | 5.92 | 1.41E-02 | 8.03 | 1.76E-04 |
| IDX_TSA_UP_CLUSTER5 | 3.65 | 7.91E-03 | 4.68 | 1.46E-05 |
| NOUZOVA_CPG_H4_UP | 2.64 | 1.90E-02 | 3.64 | 2.32E-04 |
| HSC_LATEPROGENITORS_FETAL | 4.58 | 1.28E-02 | 6.28 | 1.24E-04 |
| ROS_MOUSE_AORTA_UP | 1.67 | 3.45E-02 | 2.58 | 4.00E-04 |
| HSC_LATEPROGENITORS_SHARED | 4.63 | 1.18E-02 | 6.26 | 1.24E-04 |
| HSC_LATEPROGENITORS_ADULT | 4.62 | 1.20E-02 | 6.26 | 1.26E-04 |
| GANGLIOSIDE_BIOSYNTHESIS | 1.21 | 1.43E-02 | 1.55 | 2.67E-03 |
| AGUIRRE_PANCREAS_CHR18 | 2.06 | 4.61E-04 | 1.78 | 1.64E-03 |
| UVC_TTD_4HR_DN | 5.05 | 1.73E-02 | 6.62 | 2.23E-04 |
| UVC_XPCS_ALL_DN | 6.50 | 1.42E-02 | 8.57 | 2.09E-04 |
| BAF57_BT549_DN | 5.23 | 5.80E-03 | 6.62 | 8.45E-05 |
| HSC_MATURE_SHARED | 2.79 | 4.24E-03 | 3.19 | 2.84E-04 |
| GLOBOSIDE_METABOLISM | 1.39 | 1.51E-03 | 1.10 | 1.94E-02 |
| GOLDRATH_HP | 3.94 | 1.73E-02 | 6.34 | 3.91E-05 |
| HSC_MATURE_FETAL | 2.37 | 1.31E-02 | 2.76 | 4.61E-04 |
| AGED_MOUSE_HYPOTH_UP | 3.08 | 1.26E-02 | 4.74 | 4.26E-05 |
| HSC_INTERMEDIATEPROGENITORS_FETAL | 2.74 | 1.77E-02 | 3.43 | 3.41E-05 |
| HSA00290_VALINE_LEUCINE_AND_ISOLEUCINE_BIO… | 1.06 | 8.20E-03 | 1.69 | 2.29E-03 |
| AGED_MOUSE_RETINA_ANY_DN | 1.49 | 3.72E-03 | 2.00 | 2.23E-04 |
| FALT_BCLL_DN | 2.49 | 3.29E-02 | 3.17 | 8.47E-04 |
| HSA01032_GLYCAN_STRUCTURES_DEGRADATION | 2.60 | 1.95E-04 | 2.20 | 1.10E-04 |
| HSC_EARLYPROGENITORS_SHARED | 3.04 | 3.65E-02 | 4.96 | 2.62E-05 |
| HSC_EARLYPROGENITORS_FETAL | 3.04 | 3.65E-02 | 4.96 | 2.62E-05 |
| EIF2PATHWAY | 2.01 | 3.93E-03 | 1.19 | 1.25E-02 |
| UVC_TTD_ALL_DN | 5.33 | 1.77E-02 | 7.09 | 2.13E-04 |
| IDX_TSA_UP_CLUSTER6 | 3.56 | 1.22E-02 | 5.50 | 3.41E-05 |
| REOVIRUS_HEK293_UP | 5.59 | 7.83E-03 | 6.54 | 3.15E-04 |
| BLEO_MOUSE_LYMPH_HIGH_4HRS_UP | 2.52 | 4.03E-04 | 2.15 | 1.06E-03 |
| AGED_RHESUS_DN | 2.32 | 1.48E-02 | 3.38 | 3.06E-05 |
| HSC_INTERMEDIATEPROGENITORS_SHARED | 2.65 | 1.66E-02 | 3.44 | 3.12E-05 |
| PROTEASOME | 2.57 | 4.00E-02 | 3.33 | 3.88E-04 |
| HSA03050_PROTEASOME | 3.24 | 1.93E-02 | 4.06 | 1.02E-04 |
| HSA00562_INOSITOL_PHOSPHATE_METABOLISM | 1.77 | 9.64E-04 | 0.90 | 2.51E-02 |
| HSA00531_GLYCOSAMINOGLYCAN_DEGRADATION | 2.11 | 6.75E-04 | 1.76 | 5.09E-04 |
| HSC_EARLYPROGENITORS_ADULT | 3.16 | 3.29E-02 | 4.95 | 3.05E-05 |
| HSC_MATURE_ADULT | 3.15 | 7.78E-03 | 3.70 | 3.61E-04 |
| HOGERKORP_CD44_DN | 0.95 | 4.00E-02 | 0.88 | 3.98E-02 |
| HSC_INTERMEDIATEPROGENITORS_ADULT | 2.76 | 1.87E-02 | 3.66 | 3.41E-05 |
| GH_GHRHR_KO_24HRS_DN | 2.91 | 4.06E-02 | 4.17 | 8.56E-04 |
| JAIN_NEMO_DIFF | 1.73 | 1.38E-02 | 2.58 | 7.65E-05 |

Top 50 gene sets up-regulated in BHDS-derived tumors and also significantly higher in BHDS-derived tumors than either CH or ON tumors. Gene sets were ranked such that those with higher average enrichment score in BHDS-derived tumors are higher in the list.

**Table S4. Primer and Probe sequences for qRT-PCR validation of genes in BHD, CH, ON, and CC tumors relative to Normal kidney**

| **Gene** | **Exons** | **Assay** | **Primers/Probe** |
| --- | --- | --- | --- |
| *CDH19* | 8-9 | SYBR Green (Figure 1D) | F: ACATCAATGATCATGCTCCT  R: GATAGTCTGAATTACCTGACCA |
| *FLCN* | 8-9 | Taqman ID: Hs00376585_m1 (Figure S1A) | AGATGGAGAAGCTCGCTGATTTAGA |
| *FNIP2* | 13-14 | Taqman (Figure S1D) | As in Hasumi et al. 2008 |
| *LRRTM4* | 2-3 | SYBR Green | F: GATGACAAAGGATGGGTTTCC  R: CAGCAGTGTAGGAAGTAGCA |
| *PPARGC1A* | 1-2  12-13 | Taqman ID: Hs01016722_m1 (Figure S1G)  Taqman ID: Hs01016721_m1 (Figure S1G) | ATGGAGTGACATCGAGTGTGCTGCT  TGCAGACCTAGATTCAAACTCAGAT |
| *PVALB* | 2-3  4-5 | SYBR Green (Figure 1D)  Taqman ID: Hs00161045_m1 (Figure S1B) | F: AAAGTTCTTCCAAATGGTCG  R: TTTAGGATGAATCCCAGCTC  AAAATTGGGGTTGACGAATTCTCCA |
| *RGS20* | 4-5  5-6 | SYBR Green (Figure 1D)  Taqman ID: Hs00991569_m1 (Figure S1C) | F: CCTAAGGAGGTGAGCTTAGAC  R: GCAGAGTTCATGAATCGAGG  TCCTAAGGAGGTGAGCTTAGACTCC |
| *TFAM* | 1-2 | Taqman ID: Hs00273372_s1 (Figure 4E) | CTGGGAAGGTCTGGAGCAGAGCTGT |
| *TSC1* | 7-8 | Taqman ID: Hs00184423_m1 (Figure S1E) | AACTGGACCCTCGAAGGTGGAAGAG |

Taqman assays were ordered pre-optimized from Applied Biosystems (Foster Ciy, CA).
