# Supplementary material for: Birt-Hogg-Dubé renal tumors are genetically distinct from other renal neoplasias and are associated with up-regulation of mitochondrial gene expression
Source: BMC Med Genomics. 2010 Dec 16;3:59. doi: 10.1186/1755-8794-3-59 (PMC3012009; doi:10.1186/1755-8794-3-59)
Supplement: Additional file 2 — Supplementary Figures S1-S3. This file contains three supplementary figures: Figure S1- gene expression measurements for individual genes deregulated in BHDS tumors, Figure S2- heatmap of differentially expressed genes from Figure 1E in sporadic kidney tumors, and Figure S3- histological images of sample BHD4. [file 1755-8794-3-59-S2.PDF]

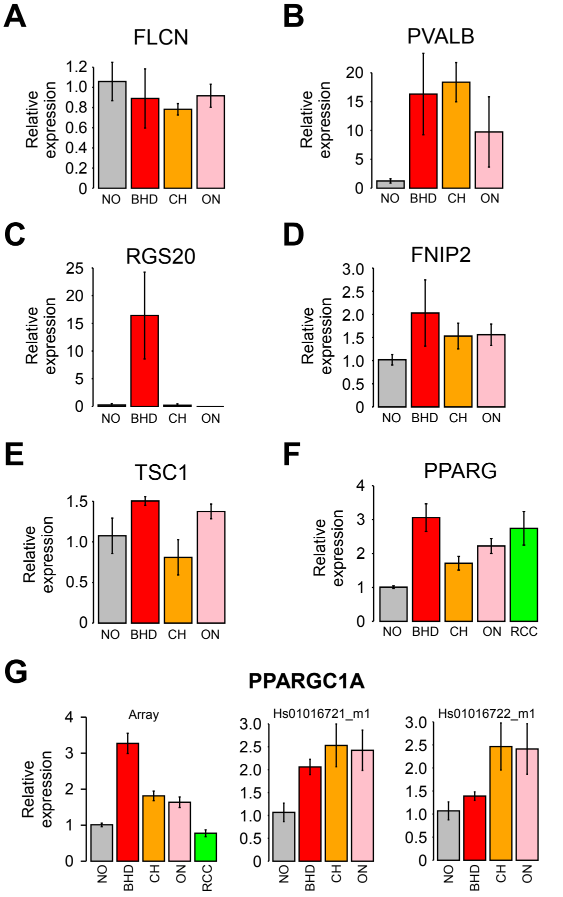

Figure S1. Gene expression of key genes deregulated in BHD, ON, and CH tumors. A-E) Validation of selected genes from gene expression microarray experiments using qRT-PCR assays (BHD, N=2; CH, N=6; ON, N=4; NO, N=4). F) Gene expression array data for PPARG in the samples identified in Figure 1. G) Gene expression array data (exon 13) and qRT-PCR validation (exons 12-13 and 1-2, respectively) for PPARGC1A. Probe and Primer information for qRT-PCR validation experiments is listed in Additional File 1, Table S4.

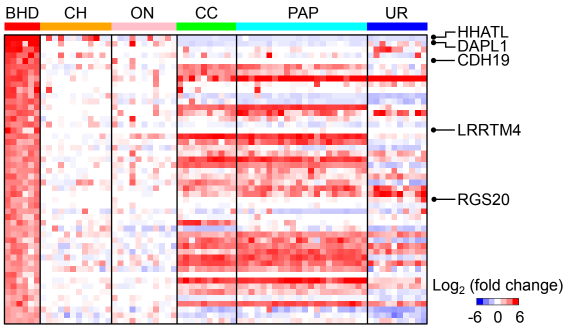

Figure S2. Genes differentially expressed in BHDS tumors compared to sporadic oncocytoma and chromophobe RCC across multiple renal tumors. Genes identified in Figure 1E are shown in clear cell, papillary, and urothelial tumors. As in Figure 1E, genes are sorted by the magnitude of differential expression between BHD as compared to CH and ON.

A

B

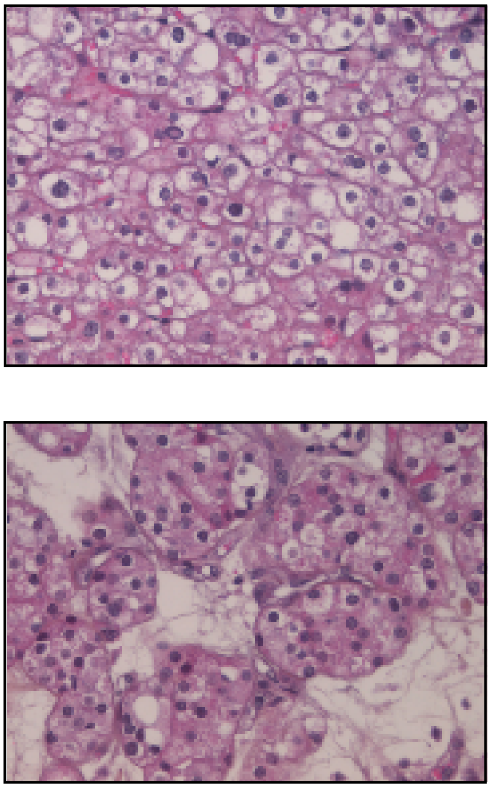

Figure S3. H&E staining to show histology of BHDS-derived hybrid oncocytic-chromophobe tumor (BHD4) with chromosomal aberrations.
A) Chromophobe-like region. B) Oncoyctic region.
